# A Re-Examination of Global Suppression of RNA Interference by HIV-1

**DOI:** 10.1371/journal.pone.0017246

**Published:** 2011-02-28

**Authors:** Viraj R. Sanghvi, Laura F. Steel

**Affiliations:** Department of Microbiology and Immunology, Institute for Molecular Medicine and Infectious Disease, Drexel University College of Medicine, Philadelphia, Pennsylvania, United States of America; Queensland Institute of Medical Research, Australia

## Abstract

The nature of the interaction between replicating HIV-1 and the cellular RNAi pathway has been controversial, but it is clear that it can be complex and multifaceted. It has been proposed that the interaction is bi-directional, whereby cellular silencing pathways can restrict HIV-1 replication, and in turn, HIV-1 can suppress silencing pathways. Overall suppression of RNAi has been suggested to occur via direct binding and inhibition of Dicer by the HIV-1 Tat protein or through sequestration of TRBP, a Dicer co-factor, by the structured TAR element of HIV-1 transcripts. The role of Tat as an inhibitor of Dicer has been questioned and our results support and extend the conclusion that Tat does not inhibit RNAi that is mediated by either exogenous or endogenous miRNAs. Similarly, we find no suppression of silencing pathways in cells with replicating virus, suggesting that viral products such as the TAR RNA elements also do not reduce the efficacy of cellular RNA silencing. However, knockdown of Dicer does allow increased viral replication and this occurs at a post-transcriptional level. These results support the idea that although individual miRNAs can act to restrict HIV-1 replication, the virus does not counter these effects through a global suppression of RNAi synthesis or processing.

## Introduction

RNA interference (RNAi) is an evolutionarily conserved mechanism of sequence dependent gene regulation that can have a role in host cell defense against intracellular pathogens and transposons. RNAi can be essential for antiviral defense in plants and lower eukaryotes (reviewed in [1]); however, in higher eukaryotes, innate immune mechanisms such as those mediated through interferons and Toll-like receptor pathways are prominent, leading to questions regarding the antiviral role of RNAi in these organisms. Nevertheless, RNAi has been implicated in restricting the replication of various mammalian viruses including HBV [2], [3], influenza A [4], and HIV-1 [5], among others.

In plants, the global suppression of RNAi by virally encoded proteins is a common countermeasure to host antiviral mechanisms mediated by RNA silencing [6], and similar suppression mechanisms have been proposed for several mammalian viruses [7], [8]. With regard to HIV-1 in particular, the HIV-1 Tat protein was reported to suppress RNAi through a direct, RNA-dependent interaction with and inhibition of Dicer [9], [10] or, alternatively, through the sequestration of mature miRNAs [11]. In addition, it has been suggested that binding of the cellular protein TRBP to the structured TAR elements present in HIV-1 transcripts competitively inhibits the activity of TRBP as a co-factor for Dicer, leading to a down-regulation of miRNA processing pathways [12], [13]. The notion that HIV-1 has evolved gene products that are able to globally suppress RNAi in order to promote its own replication has remained controversial and evidence has been presented that Tat acts only in its well-characterized role as a transcriptional activator, and does not suppress RNA silencing [14].

Multiple lines of evidence have shown that interactions between HIV-1 and cellular RNAi pathways can not only restrict HIV-1 replication but can also promote viral latency [15], [16], [17], [18], [19]. Both bioinformatics and functional studies have indicated that cellular miRNAs can affect HIV-1 replication, either through direct targeting of viral RNAs [15], [16], [17], [18], [19] or through targeting of cellular RNAs necessary for viral replication [5], [20]. Further, HIV-1 transcripts have been co-localized with RNAi effector proteins in P-bodies [18], [21], and, it has been shown that knockdown of several proteins of the miRNA processing pathway, including Dicer, Drosha, and DGCR8, leads to an increase in viral replication [5], [18]. While these results support the idea that global suppression of RNAi pathways would benefit the virus, there is also evidence indicating that HIV-1 infection, or even direct treatment of cells with Tat, is not accompanied by an overall down-regulation of miRNA expression, and instead, a more complex cellular response leads to the up- or down- regulation of individual miRNAs [5], [22], [23], [24]. In addition, HIV-1 has been successfully targeted by artificial shRNA and miRNA-based approaches (reviewed in [25], [26]) and long-term expression of therapeutic interfering RNAs has been observed in HIV-1 infected cells [27], again arguing against a significant reduction in the RNAi capacity of infected cells. While HIV-1 can escape therapeutic interfering RNAs, this has been attributed to mutations that occur in or near the target region and not to generalized inhibition of RNAi [28], [29].

Given the importance of understanding the impact of HIV-1 replication on cellular physiology in the search for vulnerabilities where antiviral therapies may be directed, including RNAi-based therapies, we have re-evaluated the potential for HIV-1 to suppress the cellular RNAi machinery. We confirm that viral replication can be limited at a post-transcriptional level by a Dicer-dependent mechanism, suggesting that the activity of certain miRNAs can reduce viral replication. However, using several measures of miRNA function and processing, we find no evidence that the virus counters these effects by suppressing RNAi through the activity of products of viral replication, including Tat and TAR.

## Results

### HIV-1 Tat protein as a suppressor of RNA silencing mediated by exogenously expressed miRNA

**Figure 1:**
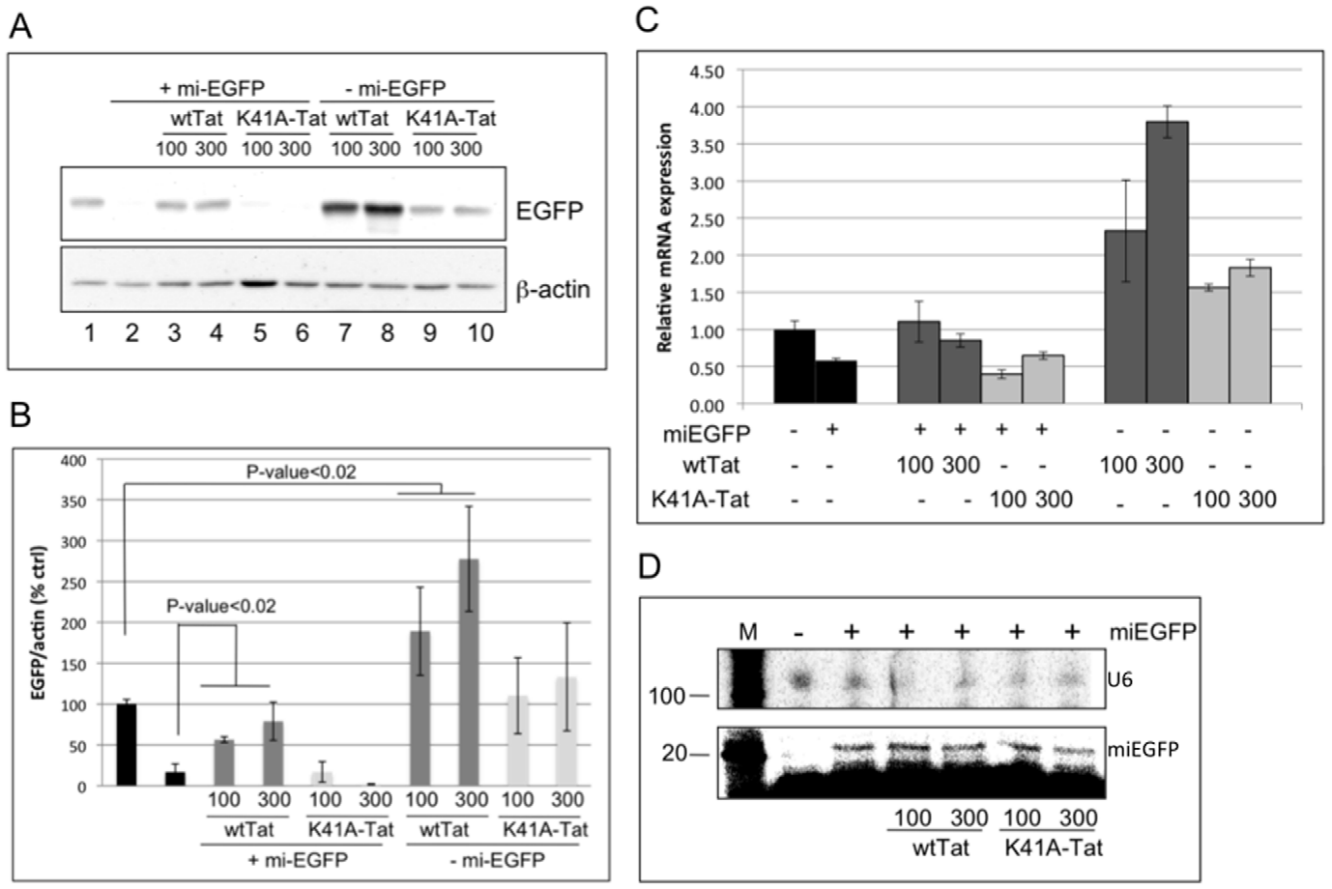
The effect of Tat over-expression on the silencing potency of miEGFP. (A) Immunoblot analysis of protein extracts obtained from P4R5 cells 2 days post-transfection with indicated plasmids. The blot was analyzed using antibodies specific to EGFP and β-actin, which serves as a loading control. (B) Spot-densitometry analysis of two individual experiments, as described in (A). The data are shown as the ratio of EGFP to β-actin and presented as the percentage of control (no miEGFP, no Tat). Error bars represents standard deviation from 3 replicates. (C) Total RNA was extracted from cells transfected in (A) and, following DNase treatment, qRT-PCR was performed to quantitate EGFP mRNA. The data are normalized to β-actin and presented as fold change over control (no miEGFP). Error bars represent standard deviation from 3 replicates. (D) Lower panel: the RNA preparation from (C) was subjected to primer extension reaction to detect mature miEGFP. Upper panel: U6 RNA was detected by northern blotting to confirm RNA integrity and quantification.

In order to understand the role of RNAi in cellular defense mechanisms against HIV-1, we first tried to confirm findings indicating that the viral Tat protein acts as a suppressor of RNA silencing (SRS). In experiments modeled after those reported by Bennasser et al. [9], we asked whether silencing of an EGFP reporter by EGFP- miRNA could be suppressed by co-expression of Tat protein. P4R5 cells, an HIV-1 reporter cell line derived from HeLa cells [30], were co-transfected with pdsEGFP and a plasmid encoding miEGFP, in the presence or absence of a Tat expression plasmid, pwtTat. As shown in Figure 1A and B, lanes 1 and 2, miEGFP was able to silence EGFP expression by approximately 80% in the absence of co-expressed Tat. In agreement with the results of Bennasser et al. [9], co-transfection with pwtTat appeared to suppress that silencing and restored EGFP expression to as much as 75% of untreated values at the highest dose (Figure 1A and B, lanes 3 and 4). However, two additional results cast doubt on the interpretation that Tat is acting to suppress silencing. First, when cells were co-transfected with pTat-K41A, a plasmid that encodes a transcriptionally inactive Tat mutant ([31] and see Figure S1), there was no rescue of EGFP expression (Figure 1A and B, lanes 5 and 6). This is in contrast to the observation by Bennasser et al. that the Tat-K41A mutant, while transcriptionally inactive, retained the ability to suppress RNA silencing [9]. Second, when cells were transfected with pwtTat in the absence of the silencing plasmid, p7SK-miEGFP, it was evident that Tat alone can substantially increase the expression of the EGFP reporter (Figure 1A and B, lanes 7 and 8). Expression of the mutant K41A Tat did not affect EGFP levels (Figure 1A and B, lanes 9 and 10). These results show a direct relationship between the transcriptional activity of Tat and its ability to up-regulate EGFP reporter expression, in a manner independent of silencing. To test further whether Tat expression up-regulates EGFP at the level of transcription rather than acting as an SRS, we used quantitative RT-PCR (qRT-PCR) to determine the level of EGFP mRNA in transfected cells from the experiment shown in Figure 1A. The results (Figure 1C) again indicate that Tat alone (in the absence of miEGFP) is acting to increase the level of EGFP mRNA, likely accounting for the increase in EGFP protein seen in cells co-transfected with the silencing plasmid, pmiEGFP, and pwtTat. These results are consistent with those reported by Lin and Cullen, who also argued against a role for Tat as a global suppressor of RNA silencing [14].

In the original report of SRS activity for Tat, evidence was presented indicating that Tat functioned by inhibiting Dicer [9], [10]. Therefore, we investigated the effect of Tat expression on the maturation of co-expressed miEGFP. Mature miEGFP, the product of Dicer cleavage of pre-miRNA [32], was assayed by primer extension of RNA isolated in Figure 1B and results are shown in Figure 1D. We found no evidence of reduced processing of pre-miEGFP in the presence of either wtTat or K41A-Tat overexpression. Control experiments showed that knockdown of Dicer by transfection of an miDicer expression plasmid resulted in reduced levels of mature miEGFP in this assay (see Figure S2).

The experiments reported above used the HeLa-derived P4R5 cell line, although the original report of Tat SRS activity used HeLa cells [9]. When we repeated the experiments in HeLa cells, we obtained very similar results to those seen with the P4R5 cells (see Figure S3). Taken together, our results support the conclusion that the apparent ability of Tat to counter the effects of silencing by miEGFP can be explained by its function as a transcriptional activator, without invoking inhibition of Dicer or suppression of silencing.

### HIV-1 Tat protein as a suppressor of RNA silencing by endogenous miRNAs

**Figure 2:**
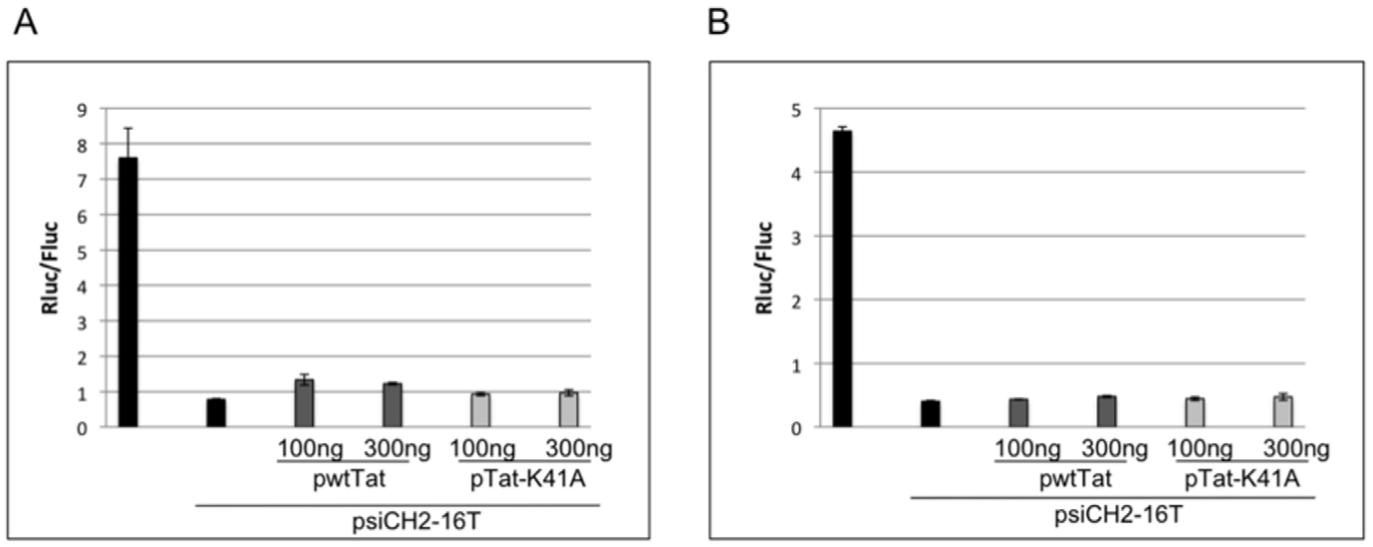
The effect of Tat over-expression on silencing by endogenous miRNA. HeLa cells (A) or P4R5 cells (B) were transfected with either psiCH2 or psiCH2-16T together with plasmid encoding wild type or mutant Tat, as indicated. The silencing efficacy of miR-16 is calculated as the ratio of Renilla luciferase (Rluc) to firefly luciferase (Fluc) activity in cell lysates 2d post-transfection. Error bars represent standard deviation from 4 replicates.

In order to test the effects of Tat expression on the activity of an endogenous cellular miRNA, we used a dual luciferase reporter vector, psiCHECK-2, with target sequence perfectly complementary to human miR-16 inserted into the 3′UTR of the Renilla luciferase (Rluc) expression cassette. The reporter plasmid, psiCH2-16T, was transfected into HeLa cells in the presence or absence of wtTat or K41A-Tat expression plasmid. Figure 2 shows that insertion of the miR-16 target into the reporter plasmid resulted in silencing of Rluc (relative to control firefly luciferase, Fluc) to about 95% of levels expressed from the reporter plasmid with no miR-16 target. This was expected, since miR-16 is readily detected in HeLa cells [33]. When the psiCH2-16T reporter plasmid was co-transfected with either pwtTat or pTat-K41A, there was no suppression of Rluc silencing. Similar results were obtained in P4R5 indicator cells, where we see no difference in silencing by miR-16 in the presence of wtTat or K41A mutant (Figure 2B). It is notable that Rluc expression is driven by an SV40 early promoter in the psiCHECK-2 vector and this promoter is minimally induced by Tat [34]. It is possible that silencing in these experiments is due to pre-existing miR-16 that could be present in sufficient amounts to suppress Rluc expression from the psiCH2-16T reporter plasmid, even if processing of new miR-16 is suppressed by Tat. However, a significant reduction in miR-16 levels has been shown in HeLa cells 48 hr after suppression of miRNA processing [35], the same time frame as our experiments. In any event, there is no evidence that the overexpression of Tat suppresses RNAi through sequestration of mature miRNAs, as suggested previously [11].

### The effects of HIV-1 replication on the cellular silencing machinery

While Tat overexpression does not affect silencing by either exogenous or endogenous miRNAs, it remained possible that other viral proteins or RNAs could alter the efficacy of RNAi. For instance, the cellular protein TRBP was originally identified based on its strong binding affinity for the HIV-1 TAR element [36] and TRBP plays a role in RNAi as a binding partner for Dicer and in recruiting Argonaute proteins to RISC [37], [38]. It has been suggested that TAR can serve to sequester TRBP and thereby reduce RNAi [12]. This idea is supported by experiments in which transfection of cells with in vitro transcribed TAR RNA hairpins led to decreased processing and activity of short hairpin (sh) RNAs and decreased processing of endogenous miRNAs [13].

**Figure 3:**
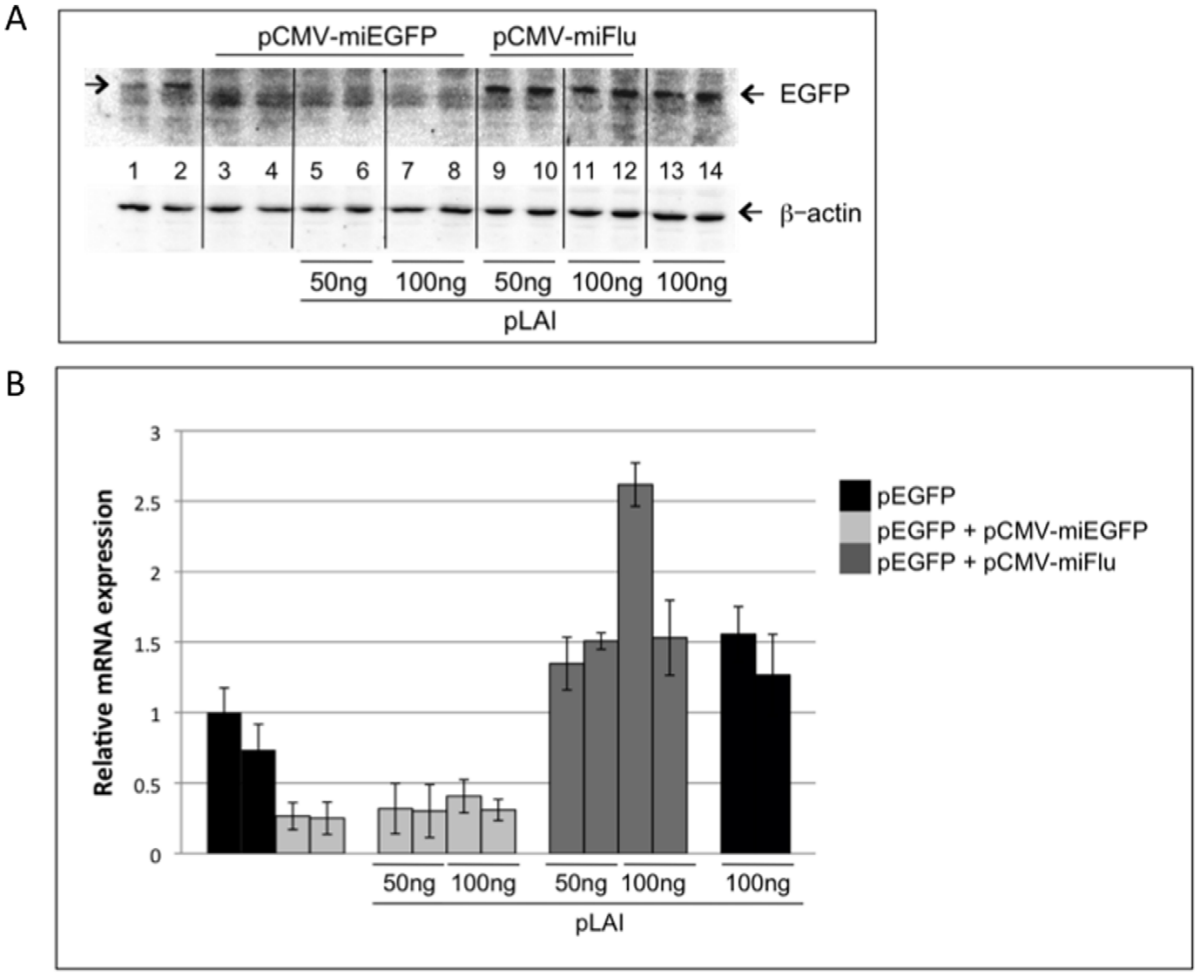
Efficacy of EGFP silencing by miEGFP in the presence of HIV-1 replication. (A) Protein extracts were prepared 2d post-transfection from 293T cells transfected with pCMV-dsEGFP and the indicated plasmids, and then analyzed by immunoblotting with antibodies specific to EGFP and β-actin. Results show two replicates from the same experiment. (B) Total RNA was isolated from cells transfected in (A) and, following DNase treatment, qRT-PCR was performed to determine the level of EGFP mRNA. The data are normalized to β-actin mRNA and presented as fold change over control. Two individual replicates from the same experiment are shown. Error bars represent standard deviation from three qPCR replicates of the same sample.

To test whether TAR present on transcripts synthesized by the replicating virus can act similarly to suppress silencing, we transfected HEK-293T cells with the HIV-1 infectious molecular clone, pLAI, and assessed silencing of EGFP reporter by a co-transfected miEGFP expression plasmid. In these experiments, the expression of miEGFP was driven by a CMV promoter, instead of the 7SK promoter used previously, to minimize effects due to differential transcriptional activation of the reporter by overexpressed Tat. As shown in Figure 3A, EGFP expression is strongly silenced by miEGFP (compare lanes 1 and 2 to lanes 3 and 4); however, there is no suppression of that silencing in the presence of replicating HIV-1 virus (compare lanes 5–8 to lanes 3 and 4). The production of infectious virus in cells transfected with pLAI was confirmed by assays of the cell culture supernatant using the P4R5 indicator cell line (data not shown). In control experiments, cells were co-transfected with a plasmid that expresses an irrelevant miRNA (pCMV-miFlu) and, as expected, these cells showed no silencing of EGFP (see Figure 3A, lanes 9–14). Notably, there was only a slight up-regulation of EGFP in the presence of virally produced Tat (compare lanes 9–14 to lanes 1 and 2 in Figure 3A), most likely due to the low levels of Tat that are produced during viral replication.

EGFP expression was further assayed at the mRNA level by qRT-PCR, and the results, shown in Figure 3B, are consistent with those obtained by immunoblotting. There is a decrease of almost 80% in EGFP mRNA in the presence of miEGFP. This is expected since the miEGFP is perfectly complementary to its target, which should lead to degradation of the EGFP mRNA. There is no suppression of silencing seen at the RNA level in the presence of replicating virus. A slight up-regulation of EGFP mRNA is observed when cells are co-transfected with pLAI and a plasmid expressing the irrelevant, control miFlu, again most likely reflecting a transcriptional response to Tat produced in these cells.

**Figure 4:**
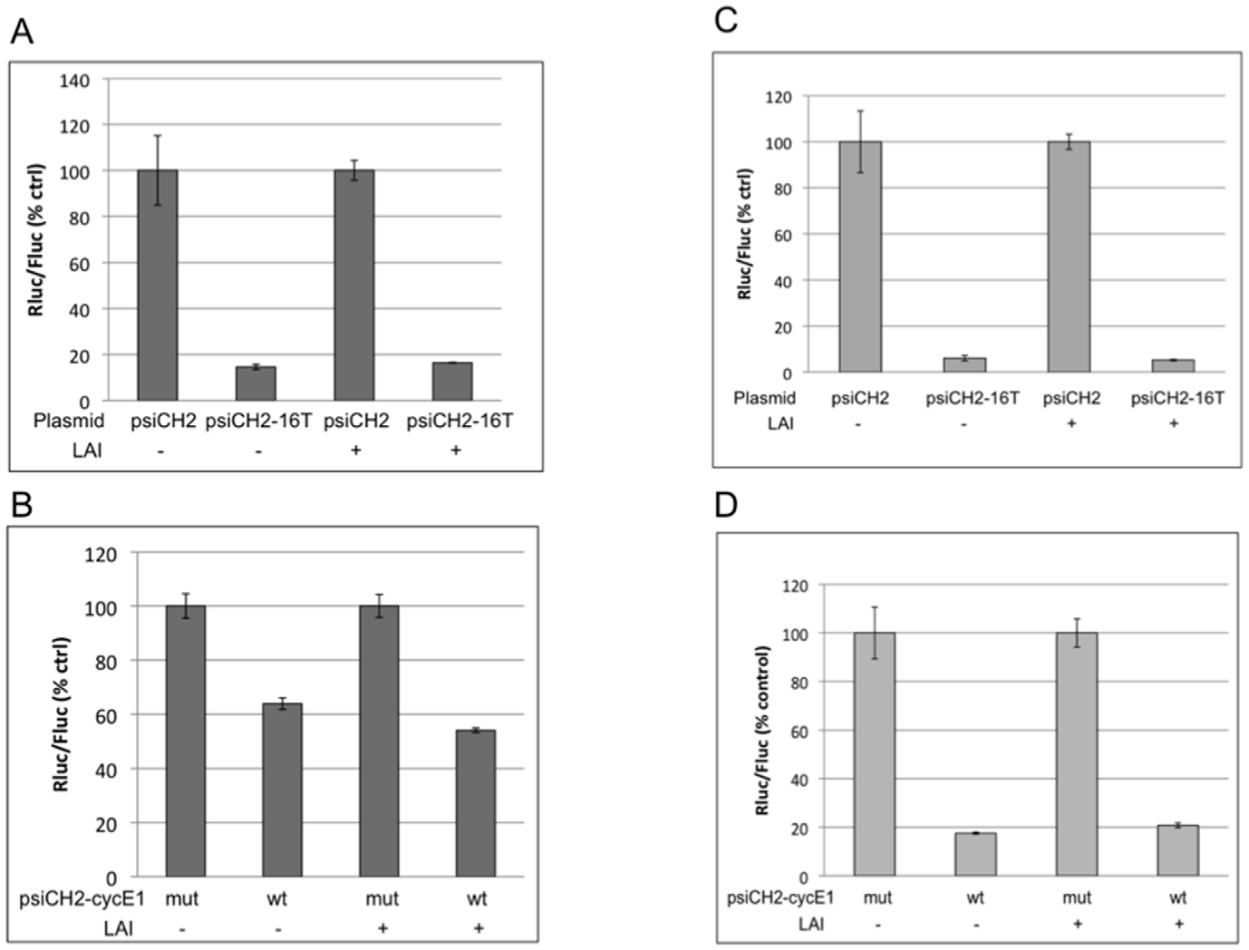
Efficacy of silencing by endogenous miR-16 in the presence of replicating HIV-1. (A) 293T cells were transfected with a reporter plasmid, either psiCH2 or psiCH2-16T, together with or without pLAI, as indicated. Cell lysates were prepared and luciferase activity was assayed 2d post-transfection. Data are normalized to the respective psiCH2 control. (B) 293T cells were transfected with either psiCH2-wtE1 or psiCH2-mutE1, in the presence or absence of pLAI, as indicated. Cells lysates were analyzed for luciferase activity as in (A). Data are normalized to the respective psiCH2-E1 control. C) Jurkat cells were transfected and analyzed for the efficacy of miR-16 silencing as in (A). D) Jurkat cells were transfected and analyzed as in (B). Error bars represent standard deviation from 4 replicates.

In the experiments shown in Figure 3, we used an interfering RNA with complete complementarity to its target site, while naturally occurring miRNAs usually show a degree of mismatch outside of the “seed” region, and this can affect their mechanism of action (reviewed in [39]). Therefore, we examined the effects of HIV-1 replication on the silencing activity of endogenous miR-16, comparing its efficacy against mRNAs with perfectly matched and imperfectly matched targets in the presence and absence of replicating virus. miR-16 has been reported to be down-regulated in HeLa cells in the presence of replicating HIV-1 or transfected TAR RNA hairpins [13], [23]. A dual luciferase reporter plasmid analogous to the psiCH2-16T reporter used in Figure 2 was constructed by inserting target sequence derived from the cyclinE1 mRNA 3′UTR. This plasmid, psiCH2-wtE1, contains two validated miR-16 target sites [40] in the Rluc 3′UTR. A plasmid with inactivating mutations in those sites [40], psiCH2-mutE1, was constructed as a control. When 293T cells were transfected with the perfectly matched target reporter, psiCH2-16T, in the presence or absence of pLAI, we observed strong silencing by endogenous miR-16 and this silencing was not suppressed by viral replication (Figure 4A), consistent with our results using miEGFP (Figure 3). Cells were then transfected with psiCH2-wtE1 and silencing of Rluc activity by miR-16 was determined by comparison to Rluc activity measured in cells transfected with the control plasmid, psiCH2-mutE1. Results (Figure 4B) again show that replicating HIV-1 does not suppress silencing by endogenous miR-16.

To test the effects of virus replication on silencing in a cell line that is more relevant to HIV-1 infection, we repeated the experiments in T-cell derived Jurkat cells. Similar to the results obtained in 293T cells, there is potent silencing by miR-16 of reporters carrying both perfectly and imperfectly matched target sites (Figure 4, C and D). In agreement with our observations in 293T cells, there was no evidence of inhibition of silencing upon HIV-1 infection.

**Figure 5:**
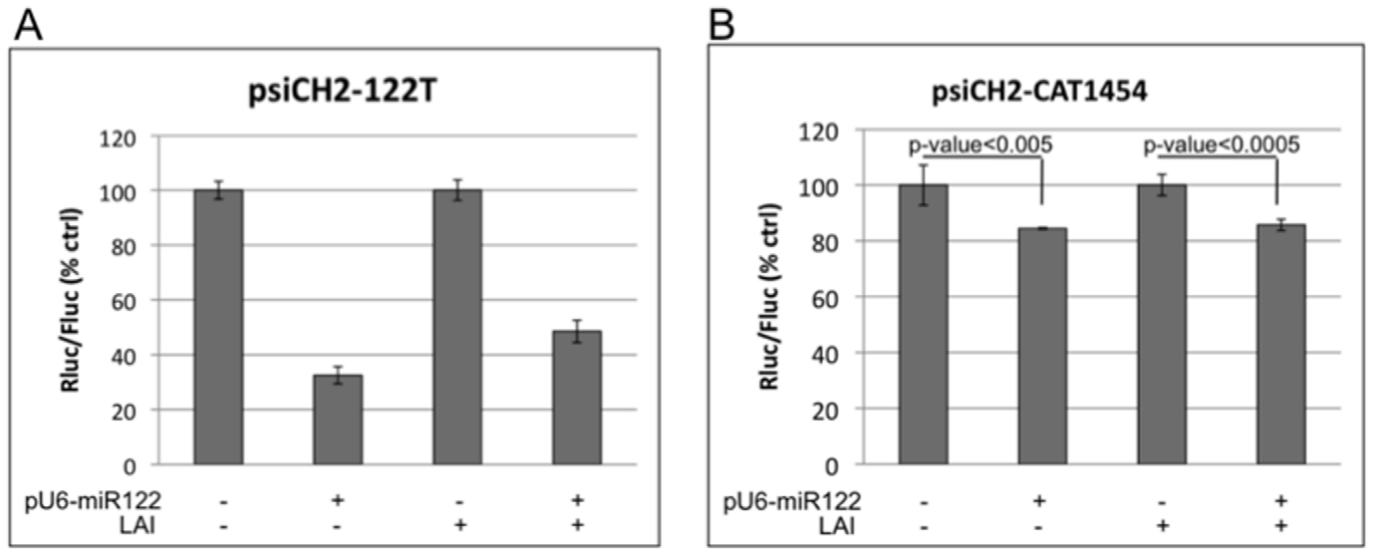
The effect of HIV-1 replication on silencing mediated by exogenously expressed miR-122. (A) 293T cells were transfected with the reporter plasmid, psiCH2-122T, with or without pU6-miR122 and pLAI as indicated. Two days post-transfection luciferase activity was assayed in cell lysates. (B) 293T cells were transfected with the reporter plasmid, psiCH2-CAT1454, with or without pU6-miR122 and pLAI as indicated. Silencing efficacy of miR-122 was analyzed 2d post-transfection, as in (A). The data are normalized to control values in respective no silencing plasmid. Error bars represent standard deviation from 4 replicates.

In order to test whether HIV-1 replication can suppress the processing or efficacy of newly synthesized miRNAs, we performed experiments similar to those above, using exogenously expressed miR-122 and reporter plasmids with either perfectly matched miR-122 target sequence or validated wild type miR-122 targets [41]. miR-122 is liver-specific and endogenous expression is undetectable in 293T cells [42]. When 293T cells were co-transfected with an miR-122 expression plasmid and the psiCH2-122T reporter, with perfectly complementary target sequence, we observed 65% silencing of Rluc activity and that silencing was not substantially suppressed in the presence of replicating virus from co-transfected pLAI (Figure 5A). Silencing of the reporter carrying validated miR-122 target sequence was not as strong (approximately 15%), but again, the potency of silencing was not affected by replicating virus (Figure 5B). In all cases, the production of infectious virus in cells transfected with pLAI was confirmed using P4R5 indicator cell assays, as above (data not shown). Overall, we find no evidence that HIV-1 replication can lead to a significant down-regulation of cellular miRNA processing or function.

### The effect of Dicer depletion on HIV-1 replication

**Figure 6:**
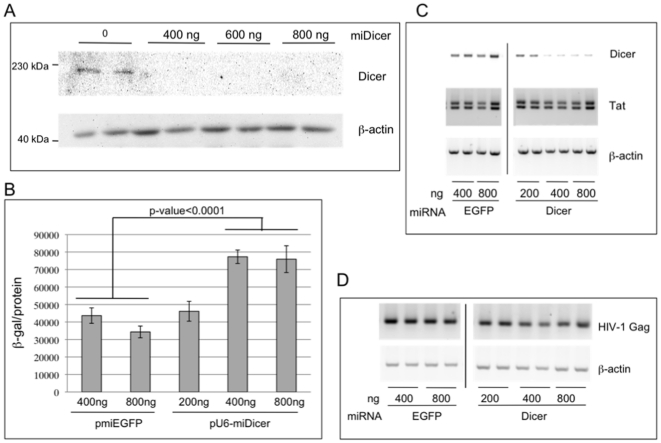
The impact of Dicer depletion on HIV-1 replication in 293T cells. (A) Dicer knockdown was confirmed by immunoblotting total protein extracts prepared 2d post-transfection from 293T cells transfected with indicated amounts of pU6-miDicer. The immunoblot was incubated with antibody specific to Dicer and β-actin that serves as a loading control. (B) 293T cells were transfected with pLAI, together with pmiEGFP or pU6-miDicer, as indicated. 2d post transfection infectious virus released into the supernatant was assayed using P4R5 indicator cells (see Materials and Methods). Error bars represent standard deviation from 6 replicates. (C) Total RNA isolated from cells in (B) was subjected to semi-quantitative RT-PCR to determine the mRNA levels of Dicer, HIV-1 Tat, and β-actin. Following PCR, products were analyzed by electrophoresis in 2% agarose and ethidium bromide staining. PCR products are resolved on the same gel and irrelevant samples are cropped out. D) Total RNA isolated in (C) was treated with DNase, and following cDNA synthesis with a Gag mRNA specific primer, semi-quantitative PCR was performed and products were analyzed as in (C). PCR for β-actin following oligo(dT)-primed cDNA synthesis serves as a loading control.

Although HIV-1 does not appear to mount a direct defense against the cellular RNAi machinery, there is considerable evidence that viral replication can be modulated by RNAi and knockdown of Dicer has been shown to up-regulate the production of infectious HIV-1 in primary cells, Jurkat cells, and 293T cells [5], [17], [18]. RNAi has been proposed to restrict HIV-1 replication either through a direct interaction between cellular miRNAs and viral transcripts, or through indirect effects on cellular proteins that act as co-factors in Tat-mediated viral transcription [5], [16]. To begin to investigate at what level Dicer-dependent RNAi acts to restrict HIV-1 replication, we compared viral RNA levels in cells with and without Dicer knockdown. First, we confirmed Dicer knockdown in 293T cells using the plasmid pmiDicer (Figure 6A), and then showed that co-transfection of this plasmid with pLAI led to an approximately 2-fold increase in the production of infectious virus (Figure 6B), consistent with published observations [18]. The release of infectious viral particles by the transfected 293T cells was measured using a P4R5 infection assay, as above. Semi-quantitative RT-PCR of RNA extracted from these cells showed that levels of Tat and Gag mRNAs are similar in cells transfected with pLAI, with or without Dicer knockdown (Figure 6C and 6D), suggesting that the rate limiting restriction to HIV-1 replication imposed by Dicer in 293T cells occurs at a post-transcriptional level. Additionally, we do not find any significant differences in the transcript levels of RNAi-related proteins upon transfection with pLAI (see Figure S4), again arguing against global regulation of the cellular RNAi machinery by products of HIV-1 infection.

## Discussion

Given the broad spectrum of host-virus interactions that can occur across the highly diverse variety of viral types, it may not be surprising that there is no consensus on the role of RNAi as an antiviral defense in mammalian cells. Instead, it appears that each virus has evolved mechanisms for avoiding the RNA silencing pathway in ways that exploit its own interactions with the physiology of its host [7], [43]. Nevertheless, it is apparent that RNAi does modulate viral replication in mammalian cells and many viruses appear to have evolved proteins or RNAs that can suppress that activity. For instance, influenza A [44], [45], vaccinia [45], Ebola [46], nodamura virus [47], HCV [48], adenovirus [49], primate foamy virus [50], and HIV-1 [9], [13] have all been reported to possess SRS activities. While adenoviral VA RNAs suppress silencing by competitively blocking Exportin-5 mediated nuclear export and Dicer processing of pre-miRNAs [49], [51], the SRS activity of several viral proteins, including NS1 of influenza A, E3L of vaccinia, VP35 of Ebola, and Tat of HIV-1, has been linked to their RNA binding domains [9], [45], [46]. Interestingly, even a bacterial dsRNA binding protein can exhibit SRS activity, suggesting that some of the viral dsRNA-binding proteins may be acting non-specifically in experiments where they are overexpressed [52]; in fact, the suppression activities of NS1, E3L, Tas, and Tat have been challenged [14], [53], [54].

Here, we show that HIV-1 Tat does not affect the silencing capacity of exogenously expressed or endogenous miRNAs, supporting the argument that this protein does not have SRS activity. Instead, our results demonstrate that the apparent SRS function of Tat is directly related to its activity as a transcriptional activator, and the up-regulation of silencing reporters can be accounted for by their increased transcription, as argued previously [14]. Our experiments extend the conclusions of Lin and Cullen by examining the function of silencing RNAs that are formatted as miRNAs instead of shRNAs [14]. Unlike shRNAs, miRNAs require the activity of the full nuclear and cytoplasmic processing pathways, involving both Drosha and Dicer, and our results indicate that Tat expression does not act as a suppressor at any point in these pathways. Further, we see no evidence of Tat-related inhibition of Dicer in direct assays of processing of exogenously expressed miRNAs.

Additional mechanisms of HIV-1 mediated RNAi suppression have been proposed, including the potential for viral TAR RNAs to compete for binding to TRBP, a co-factor for Dicer in the silencing pathway [13]. It has been reported that transfection of cells with in vitro transcribed TAR RNA can reduce the silencing potency of shRNAs as well as the processing of endogenous miRNAs [13]. However, we show that there is no reduction in the efficacy of silencing, either by exogenous or endogenous miRNAs, in the presence of replicating virus. This discrepancy might be explained if insufficient quantities of TAR RNA are present in the cytoplasm during viral replication to effectively compete for TRBP, if the cap structure or extended RNA sequence present on authentic viral transcripts can affect TRBP binding, or if a protein with redundant function in RNAi, such as PACT [55], [56], can compensate for the decreased availability of TRBP. Further, the relative affinity of TRBP for TAR, and whether it can be affected by post-translational modification in an intracellular environment, is unknown. It has been shown that TRBP can be phosphorylated, increasing its stability and enhancing RISC activity [57], and this could favor TRBP binding to RISC over binding to TAR. Clearly, much more needs to be learned regarding the complex interactions that are possible between TRBP and its potential binding partners before these effects can be explained. It has recently been shown that TAR is dispensable for viral replication when it is not required for Tat-dependent transactivation of the LTR [58]. This further supports the idea that suppression of RNAi through TAR sequestration of TRBP is not an essential viral strategy for replication.

While we find no evidence of suppression of cellular RNAi by the products of HIV-1 replication, our results do confirm that Dicer knockdown leads to increased viral replication in 293T cells. We show that this increase occurs largely at a post-transcriptional level and therefore may be due to reduced expression of miRNAs that can target viral transcripts. Our results are consistent with those of previous experiments showing that general suppression of RNAi by knockdown of Dicer in primary peripheral blood mononuclear cells (PBMCs) can lead to increased levels of viral replication in those cells [5]. The repertoire of miRNAs expressed in 293T cells is different from that of natural HIV-1 host cells, T-cells and cells of monocyte/macrophage lineage, and we cannot conclude that the mechanism of Dicer-dependent reduction of viral replication is the same in all cell types. In this regard, however, it is interesting to note that miR-382, that has been implicated in the maintenance of an unfavorable environment for HIV-1 replication in resting T-cells and in peripheral blood monocytes [16], [19], has also been identified as being particularly unstable in 293 cells after knockdown of Dicer expression [59]. Further experiments will be necessary to determine whether Dicer knockdown correlates with decreased levels of specific miRNAs that can either directly or indirectly reduce HIV-1 replication. Nevertheless, in both cultured cell models of infection and in PBMCs, the increase in viral replication under conditions of reduced levels of Dicer is only approximately 2-fold (Figure 6 and [5], [18]) and it is likely that the error prone nature of HIV-1 replication ensures that the virus does not maintain target sequence corresponding to strong antiviral cellular miRNAs.

In circumstances where Dicer activity is not limited, miRNAs can modulate the expression of cellular genes that are necessary for viral replication or pathogenesis [5], [20]. These indirect effects of miRNAs on viral replication may not be evident in the short time frame of our experiments. Although HIV-1 does not exert a global control over the activity of the cellular RNAi pathway, regulatory events triggered by viral infection and replication can result in the up- or down-regulation of the expression of numerous miRNAs [22], [23] and we expect that this plays an important role in shaping the outcome of viral infection.

## Materials and Methods

### Cell lines and plasmids

P4R5-MAGI cells (NIH AIDS Research and Reference Reagent Program #3580) were maintained in DMEM supplemented with 10% heat-inactivated FBS, sodium bicarbonate (0.05%), antibiotics (penicillin, streptomycin, and kanamycin) at 40* µ*g/mL, and puromycin (1 µg/mL). HeLa cells (ATCC #CCL-2) were maintained in the same medium as P4R5 cells but without puromycin. HEK-293T cells (ATCC #CRL-11268) were maintained in DMEM supplemented with 10% heat-inactivated FBS. Jurkat cells (ATCC #TIB-152) were grown in RPMI supplemented with 10% heat-inactivated FBS, antibiotics at 40* µ*g/mL (penicillin, streptomycin, and kanamycin), 4.5 g/L glucose, 1 mM sodium pyruvate, and 10 mM HEPES. All cells were maintained at 37°C, 5% CO_2_, and 90% relative humidity.

Construction of the plasmid encoding destabilized EGFP, pCMV-dsEGFP (pdsEGFP), was originally described by Sullivan et al. (pcDNA3.1 Neo dsEGFP APA- in [47]), and the plasmid was kindly provided by Dr. C. Sullivan. pCMV-miEGFP has been described previously [60]. p7SK-miEGFP (pmiEGFP) was constructed by removing the miEGFP encoding region from an shRNAmir construct (#RHS1706, Open Biosystems) and inserting it dowstream of a 7SK promoter in a pUC19-based vector. pU6-miDicer (pmiDicer) was similarly constructed by inserting the miDicer encoding region from an shRNAmir (#V2HS_201809, Open Biosystems) downstream of a U6 promoter in a pUC19-based vector. pCMV-wtTat (pTat) used in this study was originally constructed by Bennasser et al [9] and kindly provided by Dr. K.T. Jeang. The K41A-Tat mutant was constructed from pTat using site-directed mutagenesis (QuickChange, Stratagene). psiCH2-16T was made by inserting annealed oligonucleotides corresponding to the perfectly matched target site for human miR-16 between NotI and XhoI sites in psiCHECK2 (Promega). To construct psiCH2-wtE1, a 370 bp PCR amplicon corresponding to region 1580-1950 (GenBank NM_001238) of the cyclinE1 mRNA 3′-UTR, containing two target sites for miR-16, was inserted between NotI and XhoI sites in psiCHECK2. psiCH2-mutE1 was constructed by performing site-directed mutagenesis on psiCH2-wtE1. The construction of psiCH2-122T and psiCH2-CAT plasmids has been described previously [41]. pU6-miR122 was made by inserting a 90 bp PCR amplicon corresponding to the genomic region encoding human miR-122 downstream of a U6 promoter in a pUC19 vector. All plasmids were verified by DNA sequencing. All oligonucleotides used in these constructions are listed in Table S1.

### Transfection and reporter assays

P4R5 cells were transfected using FuGene transfection reagent (Roche) as per manufacturer's protocol. HeLa cells were transfected with GenDrill transfection reagent (BamaGen) using manufacturer's instructions. HEK-293T cells were transfected using a calcium phosphate precipitation protocol while Trans-IT Jurkat (Mirus Bio) was used to transfect Jurkat cells following manufacturer's instructions.

Renilla and firefly luciferase activities were assayed using the Dual Luciferase assay system (Promega). β-galactosidase assays were performed using the Tropix β-galactosidase assay kit (Applied Biosystems). Protein quantification to normalize the β-gal assay was done by performing Bradford assay on cell lysates (Bio-Rad).

### Immunoblots

Cell extracts were prepared for immunoblotting by lysis in 0.5X RIPA buffer supplemented with Complete Protease Inhibitor (Roche). Following treatment with benzonase nuclease (Sigma), 20-50 µg total protein was separated on 12% PAGE-SDS gel and transferred to a PVDF membrane (Millipore). Primary antibodies used in this study are ãEGFP (Chemicon), ãactin (Santa Cruz), and ãDicer (AbCam). The binding of HRP-conjugated ãRabbit IgG (Sigma) secondary antibody was detected using Super Signal West Dura Extended reagent (Pierce) and analyzed using a FluorChem digital camera (Alpha Innotech). Immunoblotting to detect Dicer was done using an 8% PAGE-SDS gel.

### P4R5 infection assay

75,000 cells were plated in a 24-well plate and incubated overnight. Cells were infected with 100 µl cell culture supernatant for 4 hours and then washed and incubated further for 2 days in fresh medium. β-galactosidase and protein assays were performed on cell lysates.

### RNA isolation, primer extension, and RT-PCR

Cells were harvested 2 days post-transfection and total RNA was isolated using TRIreagent (Sigma). For downstream applications that required DNase treatment, RNA samples were treated with Turbo DNase (Ambion). Control RT-PCRs with no added reverse transcriptase were performed to confirm complete DNA removal. For primer extension, 5 µg total RNA was incubated with a 5′-radiolabeled oligonucleotide complementary to miEGFP or Renilla luciferase mRNA at 37°C, followed by an extension reaction using M-MuLV reverse transcriptase (New England Biolabs). The extended products were resolved on a 12% polyacrylamide-TBE urea gel and detected using a Storm 820 phosphorimager (GE). U6 RNA was detected by northern blotting after separation on a 12% TBE-urea gel and transfer to a BrightStar Plus nylon membrane (Ambion). Blots were prehybridized at 37°C for 1 h in ULTRAhyb-Oligo hybridization buffer (Ambion) and then incubated overnight at 37°C with 5′-radiolabeled oligonucleotide complementary to U6 RNA. The signal was detected by phosphorimaging.

For reverse transcription, 1–2 µg total RNA was primed with oligo(dT) and reverse transcribed with M-MuLV RT. For RT-PCR of Gag mRNA, a Gag-specific primer (Table S1, HV-12) was used for priming of the reverse transcriptase reaction. The PCR products were resolved on an agarose gel and analyzed by ethidium bromide staining. Real-time PCR was done using Sybr Green chemistry (Applied Biosystems). The data were normalized to actin mRNA and analyzed using the 2^−ΔΔ_CT_^ method and SDS2.0 software (Applied Biosystems).

Sequences for primers used for these analyses are provided in Table S1.

### Statistical analysis

p-values were calculated using student's T-test.

## Supporting Information

Figure S1 **Transcriptional transactivation activity of wild type and K41A mutant Tat in P4R5 cells.** P4R5 cells were transfected with indicated amounts of wt or K41A Tat expression plasmids together with equal amounts of pRL-TK (Promega) to control for transfection efficiency. Transactivation activity of Tat is calculated as the ratio of βgal/Rluc 2d post-transfection. Error bars represent standard deviation from 4 replicates.
(TIF)

Figure S2 **Processing efficiency of pre-miEGFP to miEGFP in the absence of Dicer.** P4R5 cells were transfected with pCMV-dsEGFP and pCMV-Rluc together with pmiEGFP and pU6-miDicer, as indicated. 2 d post-transfection total RNA was isolated, treated with DNase, and subjected to primer extension reaction to determine the levels of processed miEGFP. Primer extension was also performed to detect the Renilla luciferase transcript that serves as a control for transfection efficiency.
(TIF)

Figure S3 **Effect of expression of wild type or mutant Tat on silencing by miEGFP in HeLa cells.** (A) HeLa cells were transfected with pCMV-dsEGFP, pmiEGFP, and either pwtTat or pTat-K41A, as indicated. Cell extracts were prepared 2 d post-transfection and immunoblotted to detect EGFP. β-actin was detected as a loading control. B) HeLa cells were transfected with pCMV-dsEGFP and plasmid encoding either wtTat or Tat-K41A, as indicated. EGFP and β-actin expression was analyzed by immunoblotting of cell extracts 2 d post-transfection.
(TIF)

Figure S4 **qRT-PCR analysis of mRNAs encoding key mediators of the cellular RNAi pathway upon transfection with an HIV-1 infectious molecular clone.** 293T cells were transfected with either pcDNA3.1 or pLAI as indicated and total RNA was isolated 2d post-transfection. Following reverse transcription, qPCR was performed using primers specific for Ago1, Ago2, Dicer, Drosha, and GW182. Data are normalized to β-actin mRNA and presented as fold change over levels in pcDNA3.1 transfected cells. Error bars represent standard deviation for 6 replicates.
(TIF)

Table S1 Primer sequences used in this study.
(DOC)
