# Supplementary figures and images for: A Re-Examination of Global Suppression of RNA Interference by HIV-1
Source: PLoS One. 2011 Feb 28;6(2):e17246. doi: 10.1371/journal.pone.0017246 (PMC3046114; doi:10.1371/journal.pone.0017246)

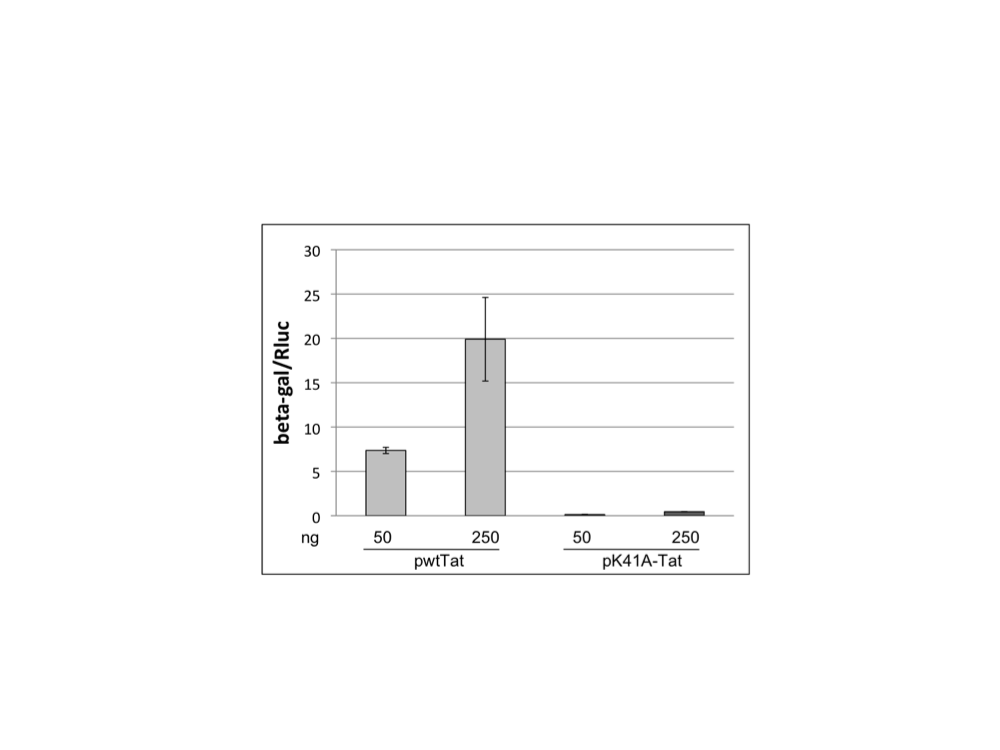

Supplement: Figure S1 — Transcriptional transactivation activity of wild type and K41A mutant Tat in P4R5 cells. P4R5 cells were transfected with indicated amounts of wt or K41A Tat expression plasmids together with equal amounts of pRL-TK (Promega) to control for transfection efficiency. Transactivation activity of Tat is calculated as the ratio of βgal/Rluc 2d post-transfection. Error bars represent standard deviation from 4 replicates. (TIF) [file pone.0017246.s001.tif]

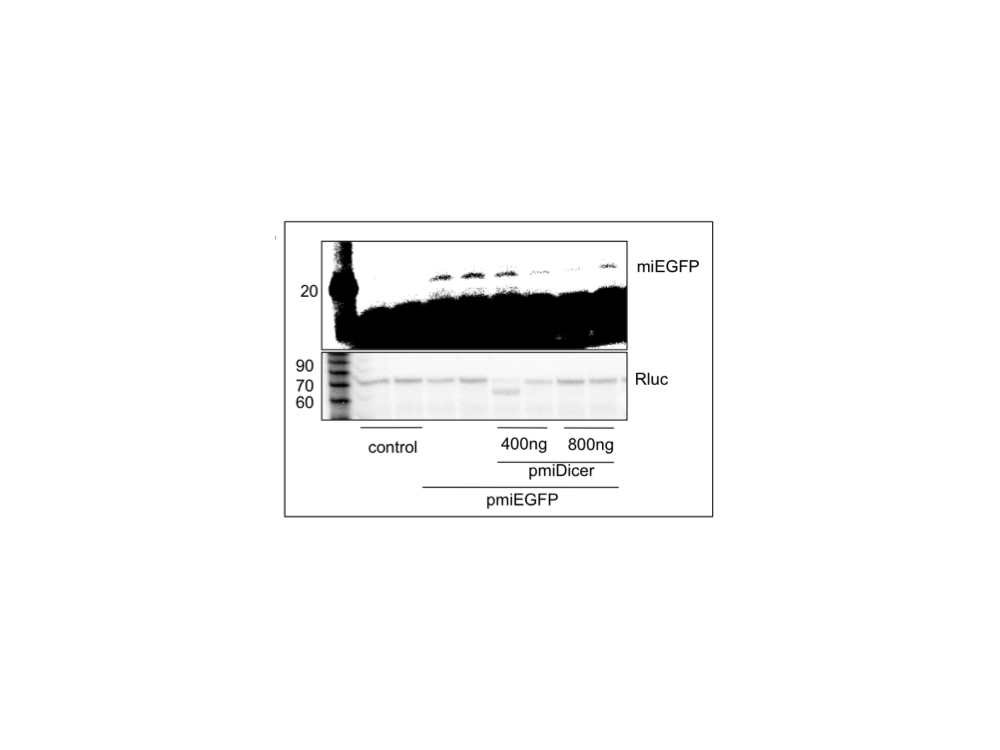

Supplement: Figure S2 — Processing efficiency of pre-miEGFP to miEGFP in the absence of Dicer. P4R5 cells were transfected with pCMV-dsEGFP and pCMV-Rluc together with pmiEGFP and pU6-miDicer, as indicated. 2 d post-transfection total RNA was isolated, treated with DNase, and subjected to primer extension reaction to determine the levels of processed miEGFP. Primer extension was also performed to detect the Renilla luciferase transcript that serves as a control for transfection efficiency. (TIF) [file pone.0017246.s002.tif]

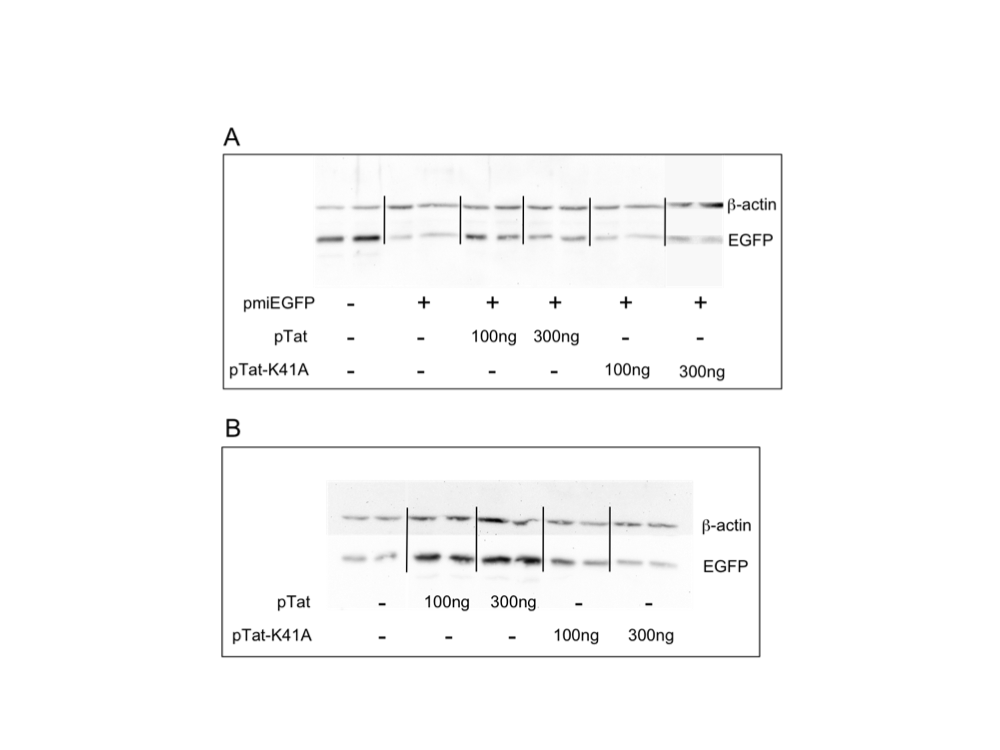

Supplement: Figure S3 — Effect of expression of wild type or mutant Tat on silencing by miEGFP in HeLa cells. (A) HeLa cells were transfected with pCMV-dsEGFP, pmiEGFP, and either pwtTat or pTat-K41A, as indicated. Cell extracts were prepared 2 d post-transfection and immunoblotted to detect EGFP. β-actin was detected as a loading control. B) HeLa cells were transfected with pCMV-dsEGFP and plasmid encoding either wtTat or Tat-K41A, as indicated. EGFP and β-actin expression was analyzed by immunoblotting of cell extracts 2 d post-transfection. (TIF) [file pone.0017246.s003.tif]

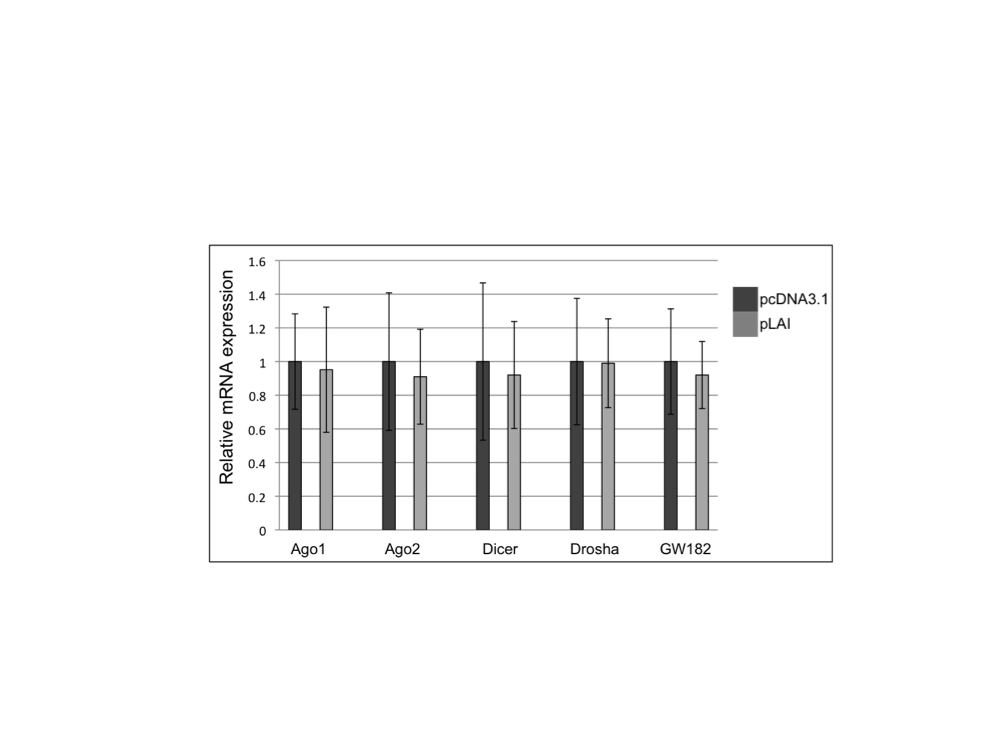

Supplement: Figure S4 — qRT-PCR analysis of mRNAs encoding key mediators of the cellular RNAi pathway upon transfection with an HIV-1 infectious molecular clone. 293T cells were transfected with either pcDNA3.1 or pLAI as indicated and total RNA was isolated 2d post-transfection. Following reverse transcription, qPCR was performed using primers specific for Ago1, Ago2, Dicer, Drosha, and GW182. Data are normalized to β-actin mRNA and presented as fold change over levels in pcDNA3.1 transfected cells. Error bars represent standard deviation for 6 replicates. (TIF) [file pone.0017246.s004.tif]
