# Supplementary material for: A Re-Examination of Global Suppression of RNA Interference by HIV-1
Source: PLoS One. 2011 Feb 28;6(2):e17246. doi: 10.1371/journal.pone.0017246 (PMC3046114; doi:10.1371/journal.pone.0017246)
Supplement: Table S1 — Primer sequences used in this study. (DOC) [file pone.0017246.s005.doc]

| Primer | Sequence (5’-3’) |
| --- | --- |
| Primers used in primer extension analysis and northern blotting | |
| PE-30 (miEGFP) | GCACAAGCTGGAGTA |
| Rluc-2 (Rluc) | GTACAGATCTCCTAGAATTACTGCTCGTTCTTCA |
| U6-4 | CTAATCCAAAAATATGGAACGCTTCACGAAT |
| Primers used for semi-quantitative RT-PCR | |
| HV-12 (Gag-RT) | GTCACTCGAGCCTGATCTCTTACCTGGCCTA |
| HV-13 (Gag-F) | GTCACTCGAGGGCCCCTAGGAAAAAGGGCTGT |
| HV-14 (Gag-R) | GTCACTCGAGAAAATTCCCGTGCCTTCCCTTGTAG |
| HV-16 (Tat-F) | CATCCCGGGTCTCTCTGGTTAGACC |
| HV-17 (Tat-R) | CGCTCTAGAAGCCATTTCTTGCTCTCCTCTG |
| Act-1 | GGACTTCGAGCAAGAGATGG |
| Act-2 | CACCTTCACCGTTCCAGTTT |
| Dcr-16 | ACAAGTACTTCAAAGCTGTCTCTCC |
| Dcr-17 | TCAGTCTAGATTGCTAGCTATTGGGAACCTGAGGTTG |
| Primers used for real-time PCR | |
| Arg-1 (Ago1-F) | GTGTCGAGAAGAGGTGCTCA |
| Arg-2 (Ago1-R) | GTTGACCCTGGATAGGCATC |
| Arg-3 (Ago2-F) | CTGTGGACACGAAAATCACC |
| Arg-4 (Ago2-R) | AGAGGACGTGATAGTGCGAA |
| Drsh-3 | CGAACCTACACTGTGGCTGT |
| Drsh-4 | CATTTCCGCTTGCTGAATAC |
| Dcr-27 | TGCAGAGGATCACTGGAATC |
| Dcr-28 | GTCTCAGTTTGGCTTCA |
| Act-3 | CCTGGCACCCAGCACAAT |
| Act-4 | GCCGATCCACACGGAGTACT |
| EGFP-8 | AGCAAAGACCCCAACGAGAA |
| EGFP-9 | GCGGCGGTCACGAAGTC |
| Primers to make psiCH2-16T | |
| Tar-9 | CGCACGCGTGGGTCTCTCTGGTTAGACCAG |
| Tar-10 | CGCACGCGTAAGCTTACATTTGCTTCTGACAC |
